# Challenges to Vaccination against SARS-CoV-2 in Patients with Immune-Mediated Diseases

**DOI:** 10.3390/vaccines9101147

**Published:** 2021-10-08

**Authors:** Giuseppe A. Ramirez, Chiara Asperti, Valentina Cucca, Mona-Rita Yacoub

**Affiliations:** 1 Division of Immunology, Transplants and Infectious Diseases, Università Vita-Salute San Raffaele, 20132 Milan, Italy; asperti.chiara@hsr.it (C.A.); vcucca82@gmail.com (V.C.); yacoub.monarita@hsr.it (M.-R.Y.); 2 Unit of Immunology, Rheumatology, Allergy and Rare Diseases, IRCCS Ospedale San Raffaele, 20132 Milan, Italy

**Keywords:** COVID-19, vaccine, autoimmune diseases, allergy, rheumatic diseases, primary immunodeficiency, mastocytosis

## Abstract

Aberrant deployment of the immune response is a hallmark pathogenic feature of severe acute respiratory syndrome coronavirus 2 (SARS-CoV-2)-related disease (COVID-19), possibly accounting for high morbidity and mortality, especially in patients with comorbidities, including immune-mediated disorders. Immunisation with SARS-COV-2 vaccines successfully instructs the immune system to limit viral spread into tissues, mitigate COVID-19 manifestations and prevent its most detrimental inflammatory complications in the general population. Patients with immune-mediated diseases have been excluded from vaccine registration trials, foreclosing the acquisition of specific efficacy and safety data. In this review, we aimed to summarise and critically discuss evidence from real-world studies addressing this issue to provide a comprehensive view of the impact of vaccination practices in patients with allergy, autoimmunity or immunodeficiency. We analysed clinical and laboratory data from 34 studies involving more than 13,000 subjects with various immune disorders who were vaccinated with mRNA- DNA- or inactivated viral particle-based vaccines. These data globally support the safe and effective use of SARS-CoV-2 vaccines in patients with immune-mediated diseases, although patient-tailored strategies to determine vaccination timing, vaccine choice and background therapy management are warranted to optimise vaccination outcomes. More data are needed regarding patients with primary immunodeficiencies.

## 1. Introduction

An embryonic concept of immunological memory had already been introduced by Thucydides in his *History of the Peloponnesian War* when describing the unique features of people surviving the V century BC Athens’ epidemic. This first notion was outlined as the ability of the host to effectively resist the most detrimental consequences of an infection after a first exposure to its aetiological agent [1]. In later centuries, the idea of uncoupling this acquired resistance from the risks of the primary infection led to the development of vaccination practices, long before the birth of modern molecular immunology [2]. Growing awareness of the physiological basis of the immune response has more recently prompted the development of increasingly accurate, safe and effective strategies for a selective stimulation of the immune system; with the purpose to elicit responsiveness towards a variety of infectious agents and possibly to non-infectious threats such as cancer cells [3]. 

As in the case of ancient Athenians, we are currently facing a pandemic crisis caused by a novel infectious agent, namely severe acute respiratory syndrome coronavirus 2 (SARS-CoV-2), which causes a multi-organ disorder characterised by systemic inflammation, prominent respiratory symptoms and relatively high rates of morbidity and mortality, conventionally referred to as coronavirus disease 2019 (COVID-19). In contrast to Thucydides’ contemporaries, we have been fortunate enough to have the scientific and industrial capacity to develop, test and clinically validate effective vaccination tools against SARS-CoV-2 in a relatively short time. Nonetheless, this age also poses novel challenges to the widespread practice of vaccination. Vaccine hesitancy is a growing issue for public health, especially in this pandemic setting. This reticent attitude may be possibly explained by a combination of factors, including (a) ever increasing expectations from Science and Medicine (also as a consequence of increasing awareness of drug and vaccine potential side effects), (b) a generalised progressive loss of trust in public institutions, and (c) misinformation [4,5].

The challenge to overcome vaccine hesitancy due to fears of potential detrimental effects of vaccination is enhanced for people living with chronic dysfunctions in the physiology of the immune response, such autoimmune/rheumatic or allergic diseases and/or primary immunodeficiencies. These subjects might in fact be at increased risk of severe or complicated COVID-19 [6,7,8] due to their constitutional and/or drug-induced immunosuppressed status. This risk is possibly more relevant for SARS-CoV-2 infection compared to other infectious diseases, since aberrant phlogistic manifestations including systemic inflammatory response syndrome and thromboinflammation are intrinsic aspects of the pathogenesis of COVID-19 even in the general population [9,10,11,12] and might be exacerbated in patients with immune-mediated diseases. Furthermore, patients with inflammatory disorders might be susceptible to flare-related morbidity either due to the direct triggering effects of viral infection [13] or to inefficient clinical/laboratory monitoring due to limitations in mobility or other containment policies [14,15]. Consequently, these patients should be (and actually have been) prioritised to vaccination, generally with a preferential indication to vaccines showing higher response rates in registration trials (such as those based on liposomal mRNA technologies). On the other hand, patients with constitutional dysfunctions of the immune response are also potentially exposed to the theoretical risk of aberrantly stimulating the immune system with a vaccine trigger. Reduced immunogenicity and clinical efficacy of vaccination due to immunosuppression/immunodeficiency constitutes an additional point of concern. Indeed, the excellent immunogenicity shown by some of the novel vaccine technologies, such as those based on mRNA, is centred on the ability of exogenous nucleic acids to prompt a robust activation of the innate immune response [3], which, however, may also play a key role in triggering inflammation in immune-mediated diseases [16,17]. In addition, most SARS-CoV-2 vaccines employ liposomal preparations conjugated with polyethylene glycol or other excipients to stabilise the mRNA-containing nanoparticles of the vaccine. These substances are associated with a relatively high prevalence of hypersensitivity reactions during parenteral administration (mostly intravenous, in contrast to the intramuscular route used for SARS-CoV-2 vaccines), even in patients with no allergy history, due to a multitude of potential mechanisms, including “true” IgE-mediated allergic responses, complement activation and/or macrophage-related immediate reactions [18,19]. Rare de novo hypersensitivity and autoimmune manifestations (including anaphylaxis, myocarditis, thrombotic thrombocytopenia and autoimmune hepatitis), have indeed been reported with multiple vaccines, in part substantiating some of the theoretical safety concerns [20,21,22,23,24]. Furthermore, although robust safety data obtained from the general population with the novel SARS-CoV-2 vaccines [25,26,27] and historical evidence with the use of non-SARS-CoV-2 vaccine in patients with immune-mediated diseases indicate that the potential benefits of vaccinating people with these disorders clearly outweigh the risks, these population subsets were excluded from SARS-CoV-2 vaccine registration trials, foreclosing any acquisition of specific data on immunological safety. Consistently, although recent surveys documented that the majority of patients had a positive attitude towards the perspective of being vaccinated with SARS-CoV-2 vaccines, the percentage of hesitant subjects was also non negligible (at least before the actual beginning of vaccination campaigns), ranging from 17% to 45% of the surveyed samples [28,29,30,31]. 

With the beginning of mass-vaccination campaigns in multiple countries, real-world data about efficacy and safety of SARS-CoV-2 vaccines in patients with immune-mediated diseases have progressively been published [32]. In this review, we aimed to summarise and discuss the current evidence on this topic and provide a comprehensive view of its potential implications for clinical practice and further research.

## 2. Methods

We interrogated the PubMed database for peer-reviewed articles published until 15 July 2021 matching the following search criteria: “COVID-19 vaccine AND autoimmune diseases”, “COVID-19 vaccine AND rheumatic diseases”, “SARS-CoV-2 vaccine AND autoimmune diseases”, “SARS-CoV-2 vaccine AND rheumatic diseases”, “COVID-19 vaccine AND allergy”, “COVID-19 vaccine AND hypersensitivity”, “SARS-CoV-2 vaccine AND allergy”, “COVID-19 vaccine AND mast-cell disease”, “SARS-CoV-2 vaccine AND mast-cell disease”, “SARS-CoV-2 vaccine AND hypersensitivity”, “COVID-19 vaccine AND primary immunodeficiency”, “SARS-CoV-2 vaccine AND primary immunodeficiency”. For aggregate analysis, we considered studies with original data only and excluded those describing single patient cases and reports of de novo autoimmune manifestations. We also excluded articles reporting duplicated data and those updated with larger samples by the same authors studying the same cohorts. For a comprehensive analysis of safety data, articles describing patients with incomplete vaccination schedules were considered separately. Works focusing on adverse events only, without data from the context cohorts, were excluded. Articles describing surrogate laboratory or clinical efficacy data in patients who did not complete their vaccination schedule or who received unscheduled supplementary vaccine doses were also excluded.

## 3. Literature Review

Thus far, several real-world observational studies, describing the clinical features and vaccination outcomes of patients with immune-mediated diseases, have been published. As of 15 July 2021, 34 studies involving more than 13,000 patients have provided data regarding efficacy/immunogenicity after complete vaccinal cycles (*n* = 21) and safety after one (*n* = 22) and two (*n* = 13) doses of SARS-CoV-2 vaccines. Most studies (33/34) included patients vaccinated with the BNT162b2 (Pfizer, *n* = 33) or the m-1372 (Moderna, *n* = 12) mRNA vaccines. Only 731 patients receiving recombinant adenoviral vector vaccines (*n* = 711 ChAdOx1-nCoV-19, Astrazeneca, *n* = 20 Ad26.COV2.S, Janssen) and 51 receiving inactivated virus vaccines (BBV152, Bharat) have been described in 7/34 studies [33,34,35,36,37,38,39]. One study reported disaggregated safety data by vaccine type, although limited to the first half of the vaccination cycle [36]. Efficacy data were available for one patient only [37]. 

Khan et al. described vaccination efficacy in a cohort of patients with IBD and reported a 93% frequency of male subjects. Most patients of other studies were women (75%). The majority of data came from subjects with an autoimmune/rheumatic disorder as primary diagnosis (84%). In 16 of 25 studies with available data, the average age of the participants was between 40 and 55 years ( Table 1 and Table 2). 

Clinical and laboratory outcomes were highly heterogeneous, reflecting the lack of consolidated data about the characteristics of the immune response in COVID-19 and anti-SARS-CoV-2 vaccination. Similar to studies investigating COVID-19 prevalence in patients with immune mediated-diseases [7,67,68,69,70], there was also broad variability in experimental methods, which ranged from web-based surveys [35,42] to clinician-supervised single- or multicentre studies [43,60] or registries [55]. Due to the recent onset of immunisation campaigns worldwide, only time-limited observations were available. Consequently, humoral and cellular immune responses to vaccination were often measured besides clinical evidence of COVID-19 cases after vaccination. Quantitative and/or qualitative assays to detect anti-SARS-CoV-2 immunoglobulins were most widely used as surrogate markers to infer vaccination responses, while only a minority of authors also investigated T cell responses. Notably, humoral and cellular responses to SARS-CoV-2 following infection or vaccination might not correlate one with each other [71,72,73] and could variably affect actual protection from eventual infections [73].

## 4. Autoimmune/Rheumatic Disorders

A total of 24 studies reporting on more than 11,000 patients with autoimmune/rheumatic diseases have been published so far. Beside one large study focused on elderly patients with inflammatory bowel diseases [55], most studies included heterogeneous patient groups with the majority of enrolled patients classified with chronic arthritides such as rheumatoid arthritis or psoriatic arthritis, in line with their relatively high prevalence in the general population (Table 2). Most patients had an established diagnosis with relatively long follow-up data. Five of seven studies where disease duration was reported, included patients living with an autoimmune disorder for an average timespan of 10–15 years [33,51,57,60,61,63]. In 12 out of 24 studies with available data (57%), the average patient age ranged between 40 and 55 years. Few, methodologically heterogeneous studies reported on comorbidities, suggesting that 34–69% of vaccinated patients with autoimmune/rheumatic diseases had one or more coexisting diseases [36,38,51,55]. Treatment data were instead reported in most studies: more than 75% of described patients were steroid-free at the time of vaccination. Conventional and biologic disease-modifying anti-rheumatic drugs were widely used by most subjects, with methotrexate (25% of patients with available data) and anti-TNF agents (22%) being the most frequently reported immunosuppressive drugs. One out of five patients was also receiving an immunomodulatory treatment with hydroxychloroquine (Supplementary Table S1). Thirteen studies addressed the question of whether SARS-CoV-2 vaccines could be associated with disease exacerbation or other adverse events in patients with autoimmune/rheumatic diseases (Table 3). Nineteen publications provided data on immunogenicity and/or short-term clinical efficacy (Table 4). 

### 4.1. Safety in Patients with Rheumatic Disorders

#### 4.1.1. Non-Disease-Related Adverse Events

Adverse events were relatively frequent after the first, second or both doses in patients with rheumatic diseases, consistent with data from the general population [25]. Among studies with safety endpoints, two deaths were reported, with no apparent correlation to vaccine administration. These events refer to a single cohort study [60] reporting a short-term fatality rate of 0.3%. The aggregate frequency of deaths among all studies with safety endpoints was 0.08%. Khan et al. [55], who described the efficacy outcomes of 6253 fully vaccinated patients with inflammatory bowel diseases, reported two additional deaths due to unspecified reasons, yielding a 0.03% mortality rate in their cohort. Hypersensitivity reactions were relatively rare (<1% incidence after the first and/or second dose), despite the relatively high prevalence of coexisting allergy history at least in some subsets of patients with autoimmune/rheumatic diseases in studies focusing on SARS-CoV-2 vaccines and in the literature [51,74,75]. Consistent with data from the general population [25], local symptoms and specifically pain at site of injection were the most frequent complaints, being reported by at least half of the described subjects. Fatigue (28%) was also highly prevalent, while other constitutional symptoms such as fever (7%), lymph-node enlargement (7%) or chilling (5%) were relatively less frequent. More than 20% of patients reported the development of headache following vaccination, and 6% had other neurological symptoms. Musculoskeletal manifestations including arthralgia/arthritis (16%) and myalgia (15%) were reported as consistently frequent adverse events in most studies. In some cases, however, they were classified as disease flares (see below). Gastrointestinal symptoms were reported by 7% of patients. Cutaneous symptoms had a relatively lower prevalence (3% after the first and/or second dose) compared to local, constitutional and musculoskeletal symptoms (Table 3). 

Five studies (three with data from subjects who completed the vaccination schedule) compared the rates of vaccination-related adverse events between patients and healthy controls, yielding conflicting results. Specifically, one study reported higher frequencies of post-vaccination symptoms in patients than in controls after the first vaccination dose [36], while two small [43,66] and two larger other studies [50,60] showed comparable or even reduced adverse event rates in patients compared to controls.

Only two studies attempted exploratory analyses to investigate potential factors associating with adverse events, and consistently reported a higher prevalence of post-vaccinal symptoms in women and younger subjects [36,51], in line with the general literature on vaccines [76]. Additional clues towards a potentially lower rate of adverse events with selected vaccines or within distinct inflammatory profiles shaped by the underlying disease and/or treatments warrant confirmation from larger studies [36,51]. 

Data regarding recombinant adenoviral vector vaccines are limited to four studies [33,35,36,38]. Three of them, including a total of 707 patients, provide information on adverse events after the first dose of the ChAdOx1-nCoV-19 (Astrazeneca) vaccine. Boekel et al. compared the clinical features of 231 patients vaccinated with ChAdOx1-nCoV-19 to those of 209 patients vaccinated with BNT162b2 and 65 with m-1372 and found an increased likelihood of reporting adverse events with the recombinant adenoviral vector vaccine [36]. Specifically, compared to patients receiving mRNA vaccines, patients vaccinated with ChAdOx1-nCoV-19 had higher rates of fever, chills, arthralgia/arthritis, fatigue, and headache. Cherian et al. and Allen-Philbey et al. did not provide disaggregated data by vaccine type, but described two heterogeneous cohorts where 87% and 88% of patients, respectively, were vaccinated with the ChAdOx1-nCoV-19 [33,38]. Consistent with the work by Boekel et al., they found a 18–21% prevalence of fever after the first vaccine dose, which is higher than reported for mRNA vaccines (Supplementary Table S2). On the other hand, relatively lower frequencies were reported for other adverse events. Due to cohort heterogeneity and absence of data from complete vaccination cycles, no definite conclusions could be driven about potential differences between adenoviral and mRNA vaccines in patients with autoimmune diseases, also in light of conflicting results in the general population [77,78]. 

#### 4.1.2. Disease Flares

In the light of concerns about the potential of vaccines to reactivate or exacerbate inflammation in patients with autoimmune disorders, disease flares were crucial parameters within safety analyses in many studies. Overall, disease flares have been reported in up to 8% of patients with autoimmune/rheumatic disorders (Table 2). However, there are several major limitations in using current evidence to draw definite conclusions about this topic. The two most significant issues are (1) limited time of post-vaccine observation (and the fact that many studies only considered features occurring after the first dose); and (2) significant discrepancies in the definition of disease flares. As an example, Barbhaiya et al. surveyed a large cohort of patients with systemic rheumatic disorders and defined a disease flare as “a sudden worsening of [patient] rheumatology condition or arthritis within 2 weeks of the vaccine”. The authors reported a 15% flare rate with predominant constitutional and joint features (“joint pain, joint swelling, muscle aches and fatigue”) and resolution within one week in 65% of cases (92% within three weeks) [35]. Similarly, Boekel et al. defined a status of “increased disease activity” as a “self-reported increase of disease activity up to two months following SARS-CoV-2 vaccination” and reported a 5% flare rate after the first dose of either mRNA or DNA-based vaccines [36], unfortunately with no follow-up data. Cherian et al. found a 0.78% flare rate after the first dose of the ChAdOx1-nCoV-19 or BBV152 vaccines, based on the use of additional non-steroidal anti-inflammatory drugs with rapid resolution requiring no changes in background therapy [38]. By contrast, other authors reported no flares after vaccination, simply because they had classified similar manifestations as (self-limited) adverse events, possibly not requiring long-term treatment changes [42,43,51,60,61,79]. Some studies also described changes in disease-specific or all-purpose activity scores after vaccination, apparently with no significant signals of concern [43,60].

### 4.2. Efficacy in Patients with Rheumatic Disorders

Of more than 8000 patients with available follow up data for an average time of 4–6 weeks after vaccination, less than 0.1% had evidence of incident COVID-19 (Table 4). Although encouraging, these data should be interpreted with extreme caution due to the limited time of observation and the lack of systematic SARS-CoV-2 testing. Consistently, most studies (17/19) also explored the dynamics of humoral and/or cellular post-vaccine immune responses in patients with autoimmune/rheumatic diseases, in a quest for potential affordable markers of immunogenicity. Taken together, patients with autoimmune/rheumatic disorders showed a good humoral and cellular response to the vaccination challenge (85% response for both parameters). Nonetheless, humoral responses including neutralisation capacity towards the receptor binding domain of SARS-CoV-2 spike protein, were quantitatively lower in patients compared to healthy controls (Table 4).

Patients receiving B-cell depleting treatments accounted for more than 10% of patients with available data (Supplementary Table S1) and constituted a subgroup of particular concern, being more susceptible to severe COVID-19 [6,80] and deemed at increased risk of no vaccine response due to the pleiotropic effects of B-cell depletion on immunogenicity. Indeed, in this subgroup of patients, markedly poor humoral response rates were observed: antibody response rates were in fact 37% aggregating data from nine studies and no publication showed positive immunoglobulins anti-SARS-CoV-2 spike protein in more than 50% of patients, including those where the median time between last B-cell depleting agent infusion and vaccination exceeded the six-month efficacy timeframe conventionally considered for rituximab and other drugs. Nonetheless, a trend towards a correlation between vaccine responsiveness and time from last immunosuppressant infusion was observed, in line with other studies [81]. 

Instead, anti-SARS-CoV-2 T-cell responses appeared relatively preserved even in patients treated with B-cell depleting agents, although the limited number of studied subjects (13/290 receiving B-cell depletion) prevents definite conclusions (Supplementary Table S3). 

Beside B-cell depletion, the use of glucocorticoids, mycophenolate, fingolimod, abatacept and multi-drug immunosuppressive therapies was also associated with lower response rates according to multiple studies [47,52,60,61]. More generally, an expected dampening effect on immunogenicity was observed with disease modifying anti-rheumatic drugs, including methotrexate [43,53]. 

## 5. Primary Immunodeficiencies

Despite evidence of increased risk of COVID-19-related morbidity and mortality in patients with primary immunodeficiencies (PID) [7], who were therefore prioritised to vaccination, data regarding vaccination outcomes in these patients are scarce. Indeed, although evidence of immunodeficiency is frequently observed in patients with autoimmune diseases (either due to intrinsic defects in the deployment of the immune response or to immune suppression), suggesting the potential reproducibility of data collected in these settings for patients with PID [82], only two studies included patients with a PID as the main diagnosis [51,58]. Regarding safety, both studies (aggregate N = 29) reported no severe adverse events following vaccination. The largest one [58], encompassing a cohort of 26 patients with various PID also documented an overall humoral and T-cellular response rate of 69% and 73%, respectively, compared to 100% response for both parameters in healthy individuals. As expected, humoral responses were impaired in patients with existing agammaglobulinemia, who, however, had satisfactory T responses, similar to pharmacologically B-depleted patients (see above). One patient with autoimmune lymphoproliferative syndrome-like disease showed neither humoral nor T cellular responses [58]. 

## 6. Allergic and Mast-Cell Disorders

More than 2100 patients with allergies with or without other immune-mediated diseases receiving anti-SARS-CoV-2 vaccination have so far been described (Table 2 and Table 5). Respiratory (prevalence 70% among allergic patients) and drug allergy (prevalence 55% among allergic patients) were the most frequent diagnoses, although patients with a history of anaphylaxis (18%) were also overrepresented compared to the general population. All studies (N = 9) included patients receiving mRNA-based vaccines; two studies also included recombinant adenoviral vector vaccines (Table 5).

No data have been published about the clinical and biological efficacy of anti-SARS-CoV-2 vaccination in patients with allergic disorders without other known immunological comorbidities. This knowledge gap might be explained by the common assumption that immunisation capacity in patients with allergic disorders should be similar to that of the general population, which however is supported by limited data only [83]. Furthermore, in slight contrast to patients with rheumatic/autoimmune diseases, patients with allergy history did not show a clearly increased risk of severe COVID-19 [84,85,86], possibly accounting for focusing on safety rather than on efficacy signals in the vast majority of studies on allergic patients. Nonetheless, patients with uncontrolled or recently exacerbated asthma, especially those with non-allergic asthma, are more susceptible to a complicated COVID-19 course [84,85,87], consistent with the known detrimental effect of viral infections to the course of asthma [88]. Assessment of vaccination efficacy in this subset of patients would be of utmost importance for current allergology practice. 

According to the currently published literature (Table 5), patients with allergy history apparently had a higher prevalence of local and systemic post-vaccination symptoms compared to subjects with no allergy [51,54]. Specifically, one large study compared the clinical features of patients with allergies receiving the BNT162b2 vaccine with those of subjects from the general population who had no allergy (but could have other comorbidities) and found that allergic subjects had higher rates of local pain (86% vs. 80% in controls), local erythema (17% vs. 11%) and local swelling (19% vs. 13%) throughout the vaccination cycle. The same study reported higher rates of systemic symptoms such as fatigue (50% vs. 45%) and arthralgia (32% vs. 26%) along with headache (42% vs. 37%), gait disturbances (2% vs. <1%), vomit (3% vs. 1%) and palpitations (7% vs. 4%) in allergic individuals compared to non-allergic individuals after the second dose of the BNT162b2 vaccine [54]. Another study, showed that patients with autoimmunity or immunodeficiency who also had an allergic background had a higher likelihood of experiencing local and constitutional symptoms with the BNT162b2 vaccine [51]. No data are currently available about the differential clinical profile of allergic subjects in comparison with proper healthy controls (although the majority of patients in ref. [54] reported no comorbidities). 

Local reactions to the first dose apparently did not associate with more severe reactions to the second dose in line with evidence from registration trials [25], and suggesting the existence of pathogenic mechanisms other than allergic sensitisation *stricto sensu* accounting for these manifestations [89]. Nonetheless, similar to patients with rheumatic disorders, allergic patients experienced adverse events more frequently after the second than after the first dose [54]. A large cohort study [54] reported that allergic manifestations were significantly more frequent in patients with a previous history of allergy than in those with no such clinical history. Nonetheless, the absolute prevalence of allergic adverse events (including anaphylaxis) was relatively low (ranging from 0% to 4%) even in populations of subjects putatively at higher risk for allergy due to their underlying disease and/or clinical history (including previous anaphylaxis; Table 5).

This result should, however, be interpreted considering the use of a variety of prophylactic strategies to minimise the risk of hypersensitivity reactions in patients with allergies. From a therapeutic point of view, the use of antihistamines and/or antileukotrienes has been reported in patients with mast-cell disorders [39,40]. Instead, there is no current evidence supporting the need for corticosteroid premedication, which could in turn dampen immunogenicity, as shown in patients with rheumatic disorders [47,52,60,61]. Beside prophylactic treatments, most authors reported the implementation of diagnostic algorithms to stratify patient risk. One approach consisted in performing pre-vaccine skin tests in all patients with a history of severe allergic reactions, regardless of a specific history suggesting sensitisation to one or more vaccine components [41]. Other authors instead triaged patients predominantly with history taking, thus limiting skin tests with either the vaccine and/or its excipients to patients with a higher pre-clinical probability of showing positive signals. With the caveat that skin tests to SARS-CoV-2 vaccines and their excipients are not standardised, data from three studies (Supplementary Table S4) reporting in vivo data show that positive skin tests to polyethylene glycol or polysorbate-80 might have a prevalence of about 1% in cohorts of patients with physician-validated diagnosis of clinically relevant allergies. This percentage might rise to about 6% in patients with definite clinical suspicion of sensitisation to vaccine excipients [34,45] and to at least 14% in patients with no previous history of allergy and new-onset hypersensitivity to the first vaccine dose (Supplementary Table S4). No patient has so far been reported with positive skin tests to one or more vaccines. Strikingly, of 226 patients who underwent a clinical/skin-test triage to confirm their fitness to safely receive anti-SARS-CoV-2 vaccination, only one (0.44%) had a mild immediate-type reaction at least after the first dose, possibly suggesting that these relatively simple approaches are feasible to apply in the normal clinical practice [41,45]. 

## 7. Conclusions

Real-world data support the safe and effective use of SARS-CoV-2 vaccines in patients with immune-mediated disorders. More extensive evidence of immunogenicity and safety has been acquired for mRNA-based vaccines compared to recombinant adenoviral vector vaccines. Taken together with efficacy signals observed in the general population, these data support the preferential use of mRNA vaccines in patients with dysregulated immune response. Despite relatively low rates of adverse events or immunisation failures among people with autoimmune/rheumatic disease, allergy or immunodeficiency, disease- and treatment-related factors might have relevant clinical implications, at least for selected patient subsets. In particular, drugs interfering with antigen presentation and B-cell biology, might impair effective humoral, but possibly not cellular immune response to vaccines, while treatments affecting other, possibly redundant inflammatory pathways, probably have less significant impacts on immunisation [90,91]. Furthermore, patients with a severe primary immunodeficiency might have insufficient vaccine responses, emphasising the need for wide vaccination campaigns in the general population. Yet, additional data from patients with primary immunodeficiencies would be particularly precious due to the current scarcity of specific evidence. Patients with a history of allergy might have a higher risk of developing vaccine-related adverse events, although most of these manifestations are expected to be mild. Conversely, disease flares in patients with rheumatic disorders appear relatively infrequent and self-limiting following vaccination, although limited evidence exists about potential risk factors for adverse vaccination outcomes in this setting. Unfavourable efficacy and safety outcomes after anti-SARS-CoV-2 vaccination might possibly be significantly attenuated by patient-centred approaches based on accurate profiling of the patients’ clinical characteristics and minimisation of immunosuppressive burden. Further studies are needed to determine the long-term efficacy and safety of anti-SARS-CoV-2 vaccination both in the general population and in patients with immune-mediated diseases. 

## Figures and Tables

**Table 1:** General features of published studies on SARS-CoV-2 vaccines in patients with immune-mediated diseases.

Ref.	Authors	First Published on	Vaccine(s)	Focus	Total pts.	Females (%)	Average Age 40–55 Years	PAID	PID	PAD	Pts. First Dose	Pts. Second Dose	Controls
[40]	Rama N et al.	19/01/2021	BNT162b2	Safety	2	2 (100)	Yes	0	0	2	2	0	0
[41]	Rojas-Pérez-Ezquerra P et al.	02/03/2021	BNT162b2 and m-1372	Safety	131	112 (85)	Yes	0	0	131	129	0	0
[42]	Connolly CM et al. *	19/03/2021	BNT162b2 and m-1372	Safety	325	312 (96)	Yes	325	0	0	325	0	0
[43]	Geisen U et al.	24/03/2021	BNT162b2 and m-1372	Efficacy and safety	26	17 (65)	Yes	26	0	0	26	26	42
[44]	Damiani G et al.	04/04/2021	BNT162b2	Efficacy and safety	4	1 (25)	Yes	4	0	0	4	4	0
[45]	Paoletti G et al.	08/04/2021	BNT162b2	Safety	414	414 (100)	ND	0	0	414	414	0	0
[34]	Banerji A et al.	15/04/2021	m1372, Ad26.COV2.S and BNT162b2	Safety	13	ND	ND	0	0	13	13	0	0
[46]	Wong S et al.	20/04/2021	BNT162b2 and m-1372	Efficacy	48	25 (52)	Yes	48	0	0	48	26	14
[47]	Achiron A et al.	22/04/2021	BNT162b2	Efficacy	125	72 (58)	ND	125	0	0	125	125	47
[37]	Buttari F et al.	04/05/2021	BNT162b2 and ChAdOx1 nCoV-19	Efficacy	4	4 (100)	ND	4	0	0	4	4	0
[48]	Dages KN et al.	05/05/2021	BNT162b2 and m-1372	Safety	68	46 (68)	Yes	0	0	68	68	0	0
[49]	Bonelli MM et al.	06/05/2021	BNT162b2	Efficacy	5	3 (60)	Yes	5	0	0	5	5	0
[50]	Simon D et al.	06/05/2021	BNT162b2	Efficacy and safety	84	55 (65)	Yes	84	0	0	84	81	182
[33]	Allen-Philbey K et al.	17/05/2021	BNT162b2 and ChAdOx1 nCoV-19	Safety	33	19 (58)	Yes	33	0	0	33	0	0
[51]	Ramirez GA et al.	24/05/2021	BNT162b2	Efficacy and safety	55	45 (82)	Yes	52	3	0 ^§^	55	55	0
[52]	Ruddy JA et al.*	24/05/2021	BNT162b2 and m-1372	Efficacy	404	385 (95)	Yes	404	0	0	404	404	0
[39]	Kaakati R et al.	24/05/2021	m1372, Ad26.COV2.S and BNT162b2	Safety	18	10 (56)	ND	0	0	18	18	18	0
[53]	Haberman RH et al.	25/05/2021	BNT162b2	Efficacy	82	58 (71)	ND	82	0	0	82	82	208
[54]	Nittner-Marszalska M et al.	25/05/2021	BNT162b2	Safety	879	ND	ND	0	0	879	879	828	0
[55]	Khan N et al.	25/05/2021	BNT162b2 and m-1372	Efficacy	7112	ND	No	7112	0	0	7112	6253	7376
[56]	Callejas-Rubio JL et al.	27/05/2021	BNT162b2	Efficacy and safety	17	12 (71)	No	17	0	0	17	17	0
[57]	Salviani C et al.	28/05/2021	BNT162b2	Efficacy	2	2 (100)	Yes	2	0	0	2	2	0
[58]	Hagin D et al.	01/06/2021	BNT162b2	Efficacy and safety	26	14 (54)	Yes	0	26	0	26	26	2
[59]	Veenstra J et al.	10/06/2021	BNT162b2 and m-1372	Efficacy	6	7 (117)	No	6	0	0	6	6	66
[60]	Furer V et al.	14/06/2021	BNT162b2	Efficacy and safety	686	475 (69)	No	686	0	0	686	686	121
[38]	Cherian S et al.	17/06/2021	ChAdOx1-nCoV-19 and BBV152	Safety	513	424 (83)	No	513	0	0	513	0	0
[61]	Braun-Moscovici Y et al.	18/06/2021	BNT162b2	Efficacy and safety	264	201 (76)	No	264	0	0	264	264	0
[36]	Boekel L et al.	18/06/2021	ChAdOx1 nCoV-19, m1372 and BNT162b2	Safety	505	329 (65)	No	505	0	0	505	73	203
[35]	Barbhaiya M et al.	22/06/2021	ChAdOx1 nCoV-19, m1372, Ad26.COV2.S and BNT162b2	Safety	1101	887 (81)	No	1101	0	0	1101	626	0
[62]	Myles IA et al.	25/06/2021	BNT162b2	Safety	581	ND	ND	0	0	581	581	581	0
[63]	Guerrieri S et al.	26/06/2021	BNT162b2 and m-1372	Efficacy	32	22 (69)	Yes	32	0	0	32	32	0
[64]	Valor-Méndez L et al.	29/06/2021	BNT162b2	Efficacy	10	8 (80)	No	10	0	0	10	10	10
[65]	Simon D et al. (2)	01/07/2021	BNT162b2	Efficacy	8	5 (63)	Yes	8	0	0	8	8	30
[66]	Mahil SK et al.	08/07/2021	BNT162b2	Efficacy and safety	84	37 (44)	Yes	84	0	0	84	0	17

ND: no data, PAD: primary allergic disease, PAID: primary autoimmune disease; PID: primary immunodeficiency. * These authors provided safety and efficacy data split in two separate study; ref. [52] was used for calculations reported in Table 2 and the text. ^§^: 22 patients had allergy as a comorbidity.

**Table 2:** Clinical features of patients with immune-mediated diseases vaccinated against SARS-CoV-2 among different studies.

	Number of Studies (%)	Number of Patients (%)
**Total number**	33 (100) *	13,344(100)
**Autoimmune disorders**	24 (73)	11,207 (84)
Connective tissue diseases	10 (30)	559 (4)
Systemic lupus erythematosus	9 (27)	307 (2)
Systemic sclerosis	4 (12)	69 (1)
Sjögren’s syndrome	4 (12)	50 (<1)
Inflammatory myopathies	8 (24)	74 (1)
Mixed connective tissue disease	4 (12)	4 (<1)
Undifferentiated connective tissue disease	1 (3)	1 (<1)
Other	1 (3)	22 (<1)
Chronic arthritides	11 (33)	1616 (12)
Rheumatoid arthritis	10 (30)	862 (6)
Psoriatic arthritis	6 (18)	267 (2)
Spondyloarthritides (other than psoriatic)	7 (21)	238 (2)
Other	4 (12)	91 (1)
Vasculitides	13 (39)	174 (1)
Large-vessel vasculitides	5 (15)	48 (<1)
Small-vessel vasculitides	7 (21)	44 (<1)
Other	3 (9)	31 (<1)
**Autoinflammatory**	5 (15)	16 (<1)
Adult-onset Still’s disease	5 (15)	8 (<1)
Other	3 (9)	9 (<1)
**Miscellanea**		
IgG4-related disease	2 (6)	8 (<1)
Psoriasis	4 (12)	79 (1)
Multiple sclerosis	6 (18)	276 (2)
Inflammatory bowel diseases	5 (15)	7172 (54)
Sarcoidosis	4 (12)	4 (<1)
Other	3 (9)	19 (<1)
**Unclassified**	NA	1286 (10)
**Primary Immunodeficiencies**	2 (6)	29 (<1)
Common variable immunodeficiency	2 (6)	14 (<1)
X-linked agammaglobulinemia	1 (3)	4 (<1)
Other	2 (6)	11 (<1)
**Allergic and mast-cell disorders**	8 (24)	2106 (16)
Anaphylaxis	3 (9)	189 (1)
Drug allergy	4 (12)	131 (1)
Respiratory allergy	3 (9)	76 (1)
Food allergy	3 (9)	53 (<1)
Hymenopter venom allergy	4 (12)	18 (<1)
Chronic spontaneous urticaria/angioedema	2 (6)	14 (<1)
Asthma	3 (9)	55 (<1)
Mastocytosis/mast-cell activation syndromes	3 (9)	21 (<1)
Other	2 (6)	22 (<1)
**Unclassified**	NA	1527 (11)

* Data from ref. [42], referring to the same cohort as ref. [52], were excluded.

**Table 3:** Safety of vaccination in patients with autoimmune diseases.

Ref.		Connolly CM et al. [42]	Geisen U et al. [43]		Allen-Philbey K et al. [33]	Ramirez GA et al. [51]	Callejas-Rubio JL et al. [56]	Furer V et al. [60]		Cherian S et al. [38]	Boekel L et al. [36]		Barbhaiya M et al. [35]	Mahil SK et al. [66]		Simon D et al. [50]		Braun-Moscovici Y et al. [61]	Damiani G et al. [44]	Total
Vaccine(s)		BNT162b2 and m-1372	BNT162b2 and m-1372		BNT162b2 and ChAdOx1 nCoV-19	BNT162b2	BNT162b2	BNT162b2		ChAdOx1-nCoV-19 and BBV152	ChAdOx1 nCoV-19, m1372 and BNT162b2		ChAdOx1 nCoV-19, m1372, Ad26.COV2.S and BNT162b2	BNT162b2		BNT162b2		BNT162b2	BNT162b2	NA
		PTS	PTS	HC	PTS	PTS	PTS	PTS	HC	PTS	PTS	HC	PTS	PTS	HC	PTS	HC	PTS	PTS	PTS
Any AE	I	ND	ND	ND	31/33 (94)	26/55 (47)	ND	ND	ND	306/513 (60)	ND	ND	ND	63/84 (75)	16/17 (94)	ND	ND	ND	3/4 (75)	429/689 (62)
	II	ND	ND	ND	ND	31/55 (56)	ND	ND	ND	ND	ND	ND	ND	ND	ND	ND	ND	ND	2/4 (50)	33/59 (56)
	Agg	ND	ND	ND	ND	38/55 (69)	ND	ND	ND	ND	ND	ND	ND	ND	ND	ND	ND	ND	3/4 (75)	41/59 (69)
Severe AEs *	I	ND	ND	ND	ND	0/55 (0)	ND	0/673 (0)	0/121 (0)	0/513 (0)	0/505 (0)	0/203 (0)	ND	0/84 (0)	0/17 (0)	0/70 (0)	0/164 (0)	ND	0/4 (0)	0/1904 (0)
	II	ND	ND	ND	ND	0/55 (0)	ND	2/670 (<1)	0/121 (0)	ND	ND	ND	ND	ND	ND	0/70 (0)	0/164 (0)	ND	0/4 (0)	2/799 (<1)
	Agg	ND	ND	ND	ND	0/55 (0)	ND	2/686 (<1)	0/121 (0)	0/513 (0)	ND	ND	ND	ND	ND	0/70 (0)	0/164 (0)	0/264 (0)	0/4 (0)	2/1592 (<1)
Allergic AEs	I	ND	ND	ND	ND	0/55 (0)	0/17 (0)	0/673 (0)	0/121 (0)	0/513 (0)	4/505 (1)	0/203 (0)	ND	ND	ND	ND	ND	ND	0/4 (0)	4/1767 (<1)
	II	ND	ND	ND	ND	2/55 (4)	0/17 (0)	1/670 (<1)	0/121 (0)	ND	ND	ND	ND	ND	ND	ND	ND	ND	0/4 (0)	3/746 (<1)
	Agg	ND	ND	ND	ND	2/55 (4)	0/17 (0)	1/686 (<1)	0/121 (0)	0/513 (0)	ND	ND	ND	ND	ND	ND	ND	ND	0/4 (0)	3/1275 (<1)
Disease flares	I	ND	0/26 (0)	NA	ND	0/55 (0)	0/17 (0)	0/673 (0)	NA	4/513 (1)	26/505 (5)	NA	117/1101 (11)	9/84 (11)	NA	ND	NA	1/264 (0)	0/4 (0)	157/3242 (5)
	II	ND	0/26 (0)	NA	ND	0/55 (0)	ND	0/673 (0)	NA	ND	ND	NA	85/626 (14)	ND	NA	ND	NA	0/264 (0)	0/4 (0)	85/1619 (5)
	Agg	ND	0/26 (0)	NA	ND	0/55 (0)	0/17 (0)	0/673 (0)	NA	ND	ND	NA	165/1101 (15)	ND	NA	ND	NA	1/264 (0)	0/4 (0)	166/2153 (8)
Local pain	I	281/325 (86)	ND	ND	23/33 (70)	16/55 (29)	ND	377/673 (56)	69/121 (57)	128/513 (25)	196/505 (39)	5/203 (1)	ND	55/84 (65)	14/17 (82)	32/70 (46)	129/164 (79)	ND	3/4 (75)	1111/2262 (49)
	II	ND	ND	ND	ND	14/55 (25)	ND	314/670 (47)	51/121 (43)	ND	ND	ND	ND	ND	ND	32/70 (46)	106/164 (65)	ND	2/4 (50)	362/799 (45)
	Agg	0/325 (0)	17/26 (65)	25/38 (66)	ND	21/55 (38)	14/17 (82)	ND	ND	ND	ND	ND	ND	ND	ND	ND	ND	ND	3/4 (75)	55/102 (54)
Fatigue	I	174/325 (54)	ND	ND	9/33 (27)	3/55 (5)	ND	4/673 (<1)	0/121 (0)	92/513 (18)	139/505 (28)	51/203 (25)	62/1101 (6)	ND	ND	14/70 (20)	48/164 (29)	ND	1/4 (25)	498/3279 (15)
	II	ND	ND	ND	ND	3/55 (5)	ND	28/670 (4)	3/121 (2)	ND	ND	ND	57/626 (9)	ND	ND	21/70 (30)	81/164 (49)	ND	0/4 (0)	109/1425 (8)
	Agg	ND	14/26 (54)	16/38 (42)	ND	5/55 (9)	5/17 (29)	ND	3/121 (2)	ND	ND	ND	ND	ND	ND	ND	ND	79/264 (30)	1/4 (25)	104/366 (28)
Arthralgia/ Arthritis	I	ND	ND	ND	ND	4/55 (7)	ND	23/673 (3)	1/121 (1)	14/513 (3)	49/505 (10)	3/203 (1)	98/1101 (9)	ND	ND	5/70 (7)	8/164 (5)	ND	0/4 (0)	193/2921 (7)
	II	ND	ND	ND	ND	7/55 (13)	ND	49/670 (7)	6/121 (5)	ND	ND	ND	74/626 (12)	ND	ND	11/70 (16)	33/164 (20)	ND	0/4 (0)	141/1425 (10)
	Agg	ND	4/26 (15)	6/38 (16)	ND	10/55 (18)	ND	ND	ND	ND	ND	ND	ND	ND	ND	ND	ND	ND	0/4 (0)	14/85 (16)
Myalgia	I	127/325 (39)	ND	ND	ND	2/55 (4)	ND	25/673 (4)	5/121 (4)	49/513 (10)	20/505 (4)	6/203 (3)	57/1101 (5)	ND	ND	6/70 (9)	17/164 (10)	ND	0/4 (0)	286/3246 (9)
	II	ND	ND	ND	ND	8/55 (15)	ND	63/670 (9)	21/121 (17)	ND	ND	ND	48/626 (8)	ND	ND	10/70 (14)	42/164 (26)	ND	0/4 (0)	129/1425 (9)
	Agg	ND	11/26 (42)	12/38 (32)	ND	10/55 (18)	ND	ND	ND	ND	ND	ND	ND	ND	ND	ND	ND	32/264 (12)	0/4 (0)	53/349 (15)
Headache	I	147/325 (45)	ND	ND	7/33 (21)	3/55 (5)	ND	47/673 (7)	7/121 (6)	71/513 (14)	124/505 (25)	45/203 (22)	ND	ND	ND	7/70 (10)	37/164 (23)	ND	0/4 (0)	406/2178 (19)
	II	ND	ND	ND	ND	6/55 (11)	ND	85/670 (13)	18/121 (15)	ND	ND	ND	ND	ND	ND	19/70 (27)	56/164 (34)	ND	0/4 (0)	122/799 (14)
	Agg	ND	10/26 (38)		ND	8/55 (15)	5/17 (29)	ND	ND	ND	ND	ND	ND	ND	ND	ND	ND	53/264 (20)	1/4 (25)	77/366 (21)
Skin rash	I	64/325 (20)	ND	ND	ND	0/55 (0)	ND	14/673 (2)	4/121 (3)	ND	22/505 (4)	5/203 (2)	14/1101 (1)	ND	ND	ND	6/164 (4)	ND	0/4 (0)	114/2663 (4)
	II	ND	ND	ND	ND	1/55 (2)	ND	10/670 (1)	6/121 (5)	ND	ND	ND	10/626 (2)	ND	ND	ND	8/164 (5)	ND	0/4 (0)	21/1355 (2)
	Agg	ND	2/26 (8)		ND	1/55 (2)	0/17 (0)	ND	ND	ND	ND	ND	ND	ND	ND	ND	ND	ND	0/4 (0)	3/102 (3)
GI symptoms	I	16/325 (5)	ND	ND	2/33 (6)	3/55 (5)	ND	12/673 (2)	1/121 (1)	ND	ND	ND	ND	ND	ND	0/70 (0)	4/164 (2)	ND	0/4 (0)	33/1160 (3)
	II	ND	ND	ND	ND	1/55 (2)	ND	17/670 (3)	2/121 (2)	ND	ND	ND	ND	ND	ND	6/70 (9)	9/164 (13)	ND	0/4 (0)	24/799 (3)
	Agg	ND	ND	ND	ND	4/55 (7)	ND	ND	ND	ND	ND	ND	ND	ND	ND	ND	ND	ND	0/4 (0)	4/59 (7)
Neurological symptoms	I	1/325 (<1)	ND	ND	4/33 (12)	3/55 (5)	ND	12/673 (2)	3/121 (2)	ND	ND	ND	ND	ND	ND	2/70 (3)	0/164 (0)	ND	0/4 (0)	22/1160 (2)
	II	ND	ND	ND	ND	6/55 (11)	ND	11/670 (2)	4/121 (3)	ND	ND	ND	ND	ND	ND	1/70 (1)	2/164 (1)	ND	0/4 (0)	18/799 (2)
	Agg	ND	ND	ND	ND	9/55 (16)	ND	ND	ND	ND	ND	ND	ND	ND	ND	ND	ND	ND	0/4 (0)	42/745 (6)
Fever	I	34/325 (10)	ND	ND	7/33 (21)	4/55 (7)	ND	8/673 (1)	1/121 (1)	94/513 (18)	56/505 (11)	21/203 (10)	6/1101 (1)	ND	ND	1/70 (1)	2/164 (1)	ND	0/4 (0)	210/3279 (6)
	II	ND	ND	ND	ND	10/55 (18)	ND	35/670 (5)	6/121 (5)	ND	ND	ND	9/626 (1)	ND	ND	7/70 (10)	16/164 (10)	ND	0/4 (0)	61/1425 (4)
	Agg	ND	0/26 (0)	0/38 (0)	ND	13/55 (24)	5/17 (29)	ND	ND	ND	ND	ND	ND	ND	ND	ND	ND	8/264 (3)	0/4 (0)	26/366 (7)
Chilling	I	45/325 (14)	ND	ND	4/33 (12)	0/55 (0)	ND	13/673 (2)	2/121 (2)	14/513 (3)	72/505 (14)	33/203 (16)	ND	ND	ND	2/70 (3)	7/164 (4)	ND	0/4 (0)	150/2178 (7)
	II	ND	ND	ND	ND	3/55 (5)	ND	60/670 (9)	21/121 (17)	ND	ND	ND	ND	ND	ND	5/70 (7)	31/164 (19)	ND	0/4 (0)	68/799 (9)
	Agg	ND	1/26 (4)	8/38 (22)	ND	3/55 (5)	ND	ND	ND	ND	ND	ND	ND	ND	ND	ND	ND	ND	0/4 (0)	4/85 (5)
Other	I	10/325 (3)	ND	ND	2/33 (6)	0/55 (0)	ND	48/673 (7)	2/121 (2)	ND	72/505 (14)	25/203 (12)	16/1101 (1)	ND	ND	8/70 (11)	5/164 (3)	ND	0/4 (0)	156/2766 (6)
	II	ND	ND	ND	ND	4/55 (7)	ND	33/670 (5)	3/121 (2)	ND	ND	ND	11/626 (2)	ND	ND	9/70 (13)	11/164 (7)	ND	0/4 (0)	57/1425 (4)
	Agg	ND	ND	ND	ND	4/55 (7)	ND	ND	ND	ND	ND	ND	27/1101 (2)	ND	ND	ND	ND	ND	0/4 (0)	31/1160 (3)

I: first dose, II: second dose, AE: adverse events, Agg: aggregate data (1st + 2nd dose), HC: healthy controls; NA: not applicable, ND: no data, PTS: patients. *: requiring hospitalisation.

**Table 4:** Efficacy of vaccination in patients with autoimmune diseases.

Ref.	Vaccine(s)	N	Clinical Endpoint (COVID-19)	Laboratory Endpoints									Responders: n(%)						Quantitative Humoral Responses (PTS vs. HC)		Main Findings
				Serology	*aSpike*	*aRBD*	*aNC*	*Neut.*	*IgA*	Cellular Responses	*IL2 ELISPOT*	*IFNγ ELISPOT*	Clinical		Humoral		T Cellular		Antibody Levels	Neutralisation Capacity	
													PTS	HC	PTS	HC	PTS	HC			
Geisen U et al. [43]	BNT162b2 and m-1372	26	No	Yes	Yes	No	No	Yes	Yes	No	No	No	ND	ND	26/26 (100)	42/42 (100)	ND	ND	2053 ± 1218 vs. 2685 ± 1182 BAU/mL	87 ± 18% vs. 96 ± 2%	3/26 patients had no IgA, antibody responses quantitatively lower in patients
Achiron A et al. [47]	BNT162b2	125	No	Yes	Yes	No	No	No	No	No	No	No	ND	ND	66/125 (53)	46/47 (98)	ND	ND	ND	ND	Impaired humoral response with fingolimod and. to a lesser extent, ocrelizumab; the humoral response was significantly affected by the time from last immunosuppressive treatment
Buttari F et al. [37]	BNT162b2 and ChAdOx1 nCoV-19	4	No	Yes	Yes	No	No	No	No	No	No	No	ND	ND	3/4 (75)	ND	ND	ND	ND	ND	Possible correlation between time from last ocrelizumab dose to vaccination and humoral response
Bonelli MM et al. [49]	BNT162b2	5	No	Yes	No	Yes	Yes	No	No	Yes	No	Yes	ND	ND	2/5 (40)	4/4 (100)	5/5 (100)	4/4 (100)	ND	ND	Anti-SARS-CoV-2 T-cell response apparently preserved and uncoupled to antibody responses (impaired in patients)
Simon D et al. [50]	BNT162b2	81	No	Yes	Yes	Yes	No	Yes	No	No	No	No	ND	ND	79/81 (98)	182/182 (100)	ND	ND	6.5± 3.1 vs. 9.4 ± 1.9 OD	ND	Neutralising humoral response in 90.5% of patients (vs 99.5% controls); potential association with immunosuppression
Ramirez GA et al. [51]	BNT162b2	52	Yes	No	No	No	No	No	No	No	No	No	52/52 (100)	ND	ND	ND	ND	ND	ND	ND	No COVID-19 cases after a median of 45 days from the II dose
Ruddy JA et al. [52]	BNT162b2 and m-1372	404	Yes	Yes	No	Yes	No	No	No	No	No	No	404/404 (100)	ND	378/404 (94)	ND	ND	ND	>250 U/mL (no control group)	ND	No COVID-19 cases at one month post-vaccination; 94% seroconversion, improving compared to first dose; rituximab, mycophenolate and glucocorticoid in association with other immunosuppressants were associated with impaired response
Haberman RH et al. [53]	BNT162b2	82	No	Yes	Yes	No	No	No	No	Yes *	No	No	ND	ND	62/82 (76)	179/179 (100)	ND	ND	113.6 (0.025–737.3) kUnits in PTS not taking MTX (N= 26) vs. 46.9 (0.025–694.5) kUnits in PTS on MTX (N=25) vs. 104.4 (0.1–601.2) kUnits in HC (N=26)	ND	Impaired humoral specific and general activated T cell response in patients on methotrexate
Callejas-Rubio JL et al. [56]	BNT162b2	17	No	Yes	Yes	No	No	Yes	No	No	No	No	ND	ND	16/17 (94)	ND	ND	ND	1205.7 (57-2080) BAU/mL (no control group)	ND	Good humoral response in a small cohort of elderly people with giant cell arteritis and mild immunosuppression
Salviani C et al. [57]	BNT162b2	2	No	Yes	Yes	No	No	No	No	No	No	No	ND	ND	0/2 (0)	ND	ND	ND	0 BAU/mL (no control group)	ND	No humoral response in two patients treated with rituximab
Veenstra J et al. [59]	BNT162b2 and m-1372	8	No	Yes	No	Yes	No	No	No	No	No	No	ND	ND	6/8 (75)	66/66 (100)	ND	ND	85.2 (29-141) vs. 178.7(163-194)-153.8 (114-194) AU/mL (age< and ≥50 years, respectively)	ND	Patients had significantly lower antibody levels than controls
Furer V et al. [60]	BNT162b2	686	Yes	Yes	Yes	No	No	Yes	No	No	No	No	686/686 (100)	120/121 (99)	590/686 (86)	121/121 (100)	ND	ND	132. 9 ± 91.7 vs. 218.6 ± 82.06 BAU	ND	No COVID-19 cases after a median of 28 days from the II dose; impaired humoral response with T-cell depletion and, to a lesser extent, with glucocorticoid, mycophenolate, abatacept and combination therapy
Braun-Moscovici Y et al. [61]	BNT162b2	264	No	Yes	No	Yes	No	No	No	No	No	No	ND	ND	227/264 (86)	ND	ND	ND	6764.3 ± 9291.6 AU/mL (no HC group)	ND	B-cell depletion, mycophenolate and abatacept were associated with lower humoral responses
Guerrieri S et al. [63]	BNT162b2 and m-1372	32	No	Yes	Yes	No	No	No	No	No	No	No	ND	ND	16/32 (50)	ND	ND	ND	ND	ND	Impaired humoral response with ocrelizumab
Valor-Méndez L et al. [64]	BNT162b2	10	No	Yes	Yes	No	No	Yes	No	No	No	No	ND	ND	9/10 (90)	10/10 (100)	ND	ND	8.4 (7.3-8.9) vs. 7.0 (6.6-7.4) OD	95.3 (87.2-96.2)% vs. 96.4 (95.4-97.2)%	Good humoral response (including neutralisation capacity) in a small cohort of patients with autoinflammatory disorders
Simon D et al. (2) [65]	BNT162b2	8	No	Yes	Yes	No	Yes	Yes	No	Yes	No	Yes	ND	ND	0/8 (0)	30/30 (100)	6/8 (75)	5/5 (100)	0.2 ± 0.3 vs. 8.1 ± 2.5 OD	ND	Impaired humoral response in COVID-naive B-cell depleted patients, despite detectable T cell responses in the majority of subjects
Khan N et al. [55]	BNT162b2 and m-1372	6253	Yes	No	No	No	No	No	No	No	No	No	6246/6253 (100)	ND	ND	ND	ND	ND	ND	ND	Two severe infections and two deaths (causes unspecified)
Damiani G et al. [44]	BNT162b2	4	No	Yes	No	Yes	No	No	No	No	No	No	ND	ND	4/4 (100)	ND	ND	ND	ND	ND	Good serological response in four patients with psoriasis treated with biologics
Wong S et al. [46]	BNT162b2 and m-1372	26	No	Yes	No	Yes	Yes	No	No	No	No	No	ND	ND	26/26 (100)	43/43 (100)	ND	ND	ND	ND	Good serological response in a series of patients with IBD
Total: N (%)	NA	8089	4/19 (21)	17/19 (89)	11/19 (58)	7/19 (37)	3/19 (16)	6/19 (32)	1/19 (5)	3/19 (16)	0/19 (0)	2/19 (11)	7388/7395 (100)	120/21 (99)	1510/1784 (85)	726/727 (100)	11/13 (85)	9/9 (100)	NA	NA	NA

aNC: anti-nucleocapside antibodies; aRBD: anti-receptor binding domain antibodies; aSpike: total anti-Spike protein antibodies; BAU: binding antibody units; MTX: methotrexate; NA: not applicable; ND: no data; Neut: neutralising antibodies; OD: optical density at ELISA testing. * Total activated T cells, (and spike-binding B cells) by flow cytometry.

**Table 5:** Safety of vaccination in patients with a history of allergy.

Ref.		Rojas-Pérez-Ezquerra P et al. [41]	Paoletti G et al. [45]	Dages KN et al. [48]	Banerji A et al. [34]	Nittner-Marszalska M et al. [54]	Rama N et al. [40]	Kaakati R et al. [39]	Myles IA et al. [62]	Ramirez GA et al. [51] §	Total
**Vaccine(s)**		BNT162b2 and m-1372	BNT162b2	BNT162b2 and m-1372	m1372, Ad26.COV2.S and BNT162b2	BNT162b2	BNT162b2	m1372, Ad26.COV2.S and BNT162b2	BNT162b2	BNT162b2	NA
**N**		131	414	68	13	879	2	18	581	22	2128
**Females: N(%)**		112 (85)	ND	46 (68)	ND	ND	2 (100)	10 (56)	ND	45 (205)	215/241 (89)
**Mean/median Age (years)**		47	ND	44	ND	ND	42	ND	ND	53	NA
**Allergy history**	**Anaphylaxis**	121 (92)	ND	ND	ND	64 (7)	ND	4 (22)	ND	ND	189/1028 (18)
	**Drug**	72 (55)	ND	32 (47)	ND	ND	ND	10 (56)	ND	17 (77)	131/239 (55)
	**Inhalants**	ND	ND	62 (91)	ND	ND	ND	6 (33)	ND	8 (36)	76/108 (70)
	**Food**	49 (37)	ND	ND	ND	ND	ND	1 (6)	ND	3 (14)	53/171 (31)
	**Hymenopters**	5 (4)	ND	8 (12)	ND	ND	ND	4 (22)	ND	1 (5)	18/239 (8)
	**Urticaria**	7 (5)	ND	ND	ND	ND	ND	ND	ND	6 (27)	13/153 (8)
	**Asthma**	45 (34)	ND	ND	ND	ND	ND	5 (28)	ND	5 (23)	55/171 (32)
	**Mastocyte diseases**	1 (1)	0 (0)	ND	ND	ND	2 (100)	18 (100)	ND	0 (0)	21/587 (4)
	**Other**	20 (15)	ND	ND	ND	ND	0 (0)	ND	ND	2 (9)	22/155 (14)
**Mean / median basal tryptase (ng/mL)**		ND	ND	ND	ND	ND	14,3	44,6	0	ND	NA
**Any AE**	**I**	1 (1)	ND	0 (0)	ND	817 (93)	1 (50)	ND	ND	10 (45)	829/1102 (75)
	**Median time to onset (h)**	0.16	ND	NA	ND	ND	24	ND	ND	24	NA
	**Median time to resolution (h)**	0.16	ND	NA	ND	48	24	ND	ND	48	NA
	**II**	ND	ND	ND	ND	762 (92)	ND	ND	ND	17 (77)	779/901 (86)
	**Median time to onset (h)**	ND	ND	ND	ND	ND	ND	ND	ND	24	NA
	**Median time to resolution (h)**	ND	ND	ND	ND	48	ND	ND	ND	48	NA
	Agg	ND	ND	ND	ND	ND	ND	ND	ND	18 (82)	18/22 (82)
Severe AEs *	**I**	0 (0)	0 (0)	0 (0)	ND	ND	0 (0)	ND	0 (0)	0 (0)	0/1218 (0)
	**II**	ND	ND	ND	ND	ND	ND	ND	0 (0)	0 (0)	0/603 (0)
	Agg	ND	ND	ND	ND	ND	ND	ND	0 (0)	0 (0)	0/603 (0)
**Allergic AEs**	**I**	1 (1)	ND	0 (0)	0 (0)	30 (3)	ND	0 (0)	0 (0)	0 (0)	31/1712 (2)
	**II**	ND	ND	ND	ND	33 (4)	ND	0 (0)	0 (0)	0 (0)	33/1449 (2)
	Agg	ND	ND	ND	ND	33 (4)	ND	0 (0)	0 (0)	0 (0)	33/1449 (2)
**Anaphylaxis**	**I**	0 (0)	0 (0)	0 (0)	ND	ND	0 (0)	0 (0)	0 (0)	0 (0)	0/1236 (0)
	**II**	ND	ND	ND	ND	ND	ND	0 (0)	0 (0)	0 (0)	0/621 (0)
	Agg	ND	ND	ND	ND	ND	ND	ND	0 (0)	0 (0)	0/603 (0)
**Local pain**	**I**	ND	ND	ND	ND	714 (81)	0 (0)	ND	ND	9 (41)	723/903 (80)
	**II**	ND	ND	ND	ND	643 (78)	ND	ND	ND	9 (41)	652/850 (77)
**Fatigue**	**I**	ND	ND	ND	ND	ND	0 (0)	ND	ND	0 (0)	0/24 (0)
	**II**	ND	ND	ND	ND	436 (53)	ND	ND	ND	1 (5)	437/850 (51)
**Arthralgia /Arthritis**	**I**	ND	ND	ND	ND	80 (9)	0 (0)	ND	ND	1 (5)	81/903 (9)
	**II**	ND	ND	ND	ND	279 (34)	ND	ND	ND	5 (23)	284/850 (33)
**Myalgia**	**I**	ND	ND	ND	ND	155 (18)	1 (50)	ND	ND	0 (0)	156/903 (17)
	**II**	ND	ND	ND	ND	421 (51)	ND	ND	ND	4 (18)	425/850 (50)
**Headache**	**I**	ND	ND	ND	ND	149 (17)	0 (0)	ND	ND	1 (5)	150/903 (17)
	**II**	ND	ND	ND	ND	368 (44)	ND	ND	ND	3 (14)	371/850 (44)
**Neurological symptoms**	**I**	ND	ND	ND	ND	21 (2)	0 (0)	ND	ND	1 (5)	22/903 (2)
	**II**	ND	ND	ND	ND	ND	ND	ND	ND	3 (14)	3/22 (14)
**Fever**	**I**	ND	ND	ND	ND	13 (1)	0 (0)	ND	ND	2 (9)	15/903 (2)
	**II**	ND	ND	ND	ND	142 (17)	ND	ND	ND	6 (27)	148/850 (17)

I: first dose, II: second dose, AE: adverse events, Agg: aggregate data (1st + 2nd dose), NA: not applicable, ND: no data. *: requiring hospitalisation. §: patients with allergy as a comorbidity of either primary immunodeficiency or rheumatic disease.

## Data Availability

The authors can share the supporting data upon reasonable requests to the Corresponding Author.

## Supplementary Materials

The following are available online at https://www.mdpi.com/article/10.3390/vaccines9101147/s1, Table S1: immunosuppressive treatments in patients with autoimmune diseases; Table S2: selected adverse reactions after the first dose of adenoviral-vectored and mRNA vaccines in patients with autoimmune diseases; Table S3: vaccination efficacy in patients treated with B-cell depleting agents; Table S4: incidence of positive skin tests to vaccine excipient in patients with allergy history and incident allergy to anti-SARS-CoV-2 vaccines
