# Supplementary material for: Challenges to Vaccination against SARS-CoV-2 in Patients with Immune-Mediated Diseases
Source: Vaccines (Basel). 2021 Oct 8;9(10):1147. doi: 10.3390/vaccines9101147 (PMC8537812; doi:10.3390/vaccines9101147)
Supplement: Supplementary file 1 [file vaccines-09-01147-s001.zip › vaccines-1386981-supplementary.pdf]

Supplementary Table 1: immunosuppressive treatments in patients with autoimmune diseases

Ref.	N	PDN	Mean/ Median PDN dose (mg)	HCQ	MMF	B-cell depletion	RTX	MTX	AZA	aTNF	aIL6	Colchicine	OCZ	Other
Geisen U et al	26	7 (27)	5	3 (12)	0 (0)	0 (0)	0 (0)	0 (0)	1 (4)	12 (46)	3 (12)	0 (0)	0 (0)	5 (19)
Achiron A et al	125	ND	ND	0 (0)	0 (0)	44 (35)	0 (0)	0 (0)	0 (0)	0 (0)	0 (0)	0 (0)	44 (35)	49 (39)
Buttari F et al.	4	ND	ND	0 (0)	0 (0)	2 (50)	0 (0)	0 (0)	0 (0)	0 (0)	0 (0)	0 (0)	2 (50)	1 (25)
Bonelli MM et al.	5	3 (60)	ND	0 (0)	2 (40)	5 (100)	5 (100)	1 (20)	0 (0)	0 (0)	0 (0)	0 (0)	0 (0)	1 (20)
Simon D et al.	84	10 (12)	ND	3 (4)	0 (0)	0 (0)	0 (0)	16 (19)	0 (0)	11 (13)	3 (4)	0 (0)	0 (0)	23 (27)
Allen-Philbey K et al.	33	ND	ND	0 (0)	0 (0)	17 (52)	1 (3)	0 (0)	0 (0)	0 (0)	0 (0)	0 (0)	15 (45)	11 (33)
Ramirez GA et al.	52	20 (38)	5	18 (35)	5 (10)	6 (12)	6 (12)	15 (29)	2 (4)	12 (23)	4 (8)	5 (10)	0 (0)	10 (19)
Ruddy JA et al.	404	117 (29)	ND	170 (42)	41 (10)	19 (5)	19 (5)	94 (23)	35 (9)	98 (24)	7 (2)	0 (0)	0 (0)	168 (42)
Haberman RH et al.	82	ND	ND	3 (4)	0 (0)	1 (1)	1 (1)	45 (55)	0 (0)	31 (38)	ND	0 (0)	0 (0)	16 (20)
Callejas-Rubio JL et al	17	9 (53)	5	0 (0)	0 (0)	0 (0)	0 (0)	1 (6)	0 (0)	0 (0)	3 (18)	0 (0)	0 (0)	0 (0)
Salviani C et al.	2	1 (50)	3.8	0 (0)	0 (0)	2 (100)	2 (100)	0 (0)	0 (0)	0 (0)	0 (0)	0 (0)	0 (0)	0 (0)
Veenstra J et al.	6	2 (33)	5	1 (17)	1 (17)	0 (0)	0 (0)	2 (33)	1 (17)	1 (17)	0 (0)	0 (0)	0 (0)	1 (17)
Furer V et al.	686	130 (19)	6.7	133 (19)	28 (4)	87 (13)	86 (13)	176 (26)	0 (0)	172 (25)	37 (5)	0 (0)	ND	114 (17)
Cherian S et al.	513	97 (19)	ND	ND	ND	ND	ND	ND	ND	ND	ND	ND	ND	ND
Braun-Moscovici Y et al.	264	92 (35)	ND	43 (16)	26 (10)	48 (18)	ND	78 (30)	14 (5)	63 (24)	ND	ND	ND	ND
Boekel L et al.	505	77 (15)	ND	ND	ND	35 (7)	ND	169 (33)	ND	93 (18)	1 (0)	ND	ND	115 (23)
Barbhaiya M et al.	1101	ND	ND	ND	ND	ND	ND	ND	ND	ND	ND	ND	ND	ND
Guerrieri S et al.	32	ND	ND	ND	ND	16 (50)	ND	ND	ND	ND	ND	ND	16 (50)	16 (50)
Valor-Méndez L et al.	10	0 (0)	0	0 (0)	0 (0)	0 (0)	0 (0)	0 (0)	0 (0)	0 (0)	0 (0)	0 (0)	0 (0)	10 (100)
Simon D et al. (2)	8	ND	ND	ND	ND	8 (100)	8 (100)	ND	ND	ND	ND	ND	0 (0)	ND
Mahil SK et al.	84	0 (0)	0	0 (0)	0 (0)	0 (0)	0 (0)	17 (20)	0 (0)	27 (32)	0 (0)	0 (0)	0 (0)	25 (30)
Khan N et al.	7,112	ND	ND	ND	ND	ND	ND	ND	ND	ND	ND	ND	ND	ND
Damiani G et al.	4	0 (0)	0	0 (0)	0 (0)	0 (0)	0 (0)	0 (0)	0 (0)	0 (0)	0 (0)	0 (0)	0 (0)	4 (100)
Wong S et al.	26	0 (0)	0	0 (0)	0 (0)	0 (0)	0 (0)	0 (0)	0 (0)	8 (31)	0 (0)	0 (0)	0 (0)	14 (54)
Total	11,207	565/2710 (21)	NA	374/1936 (19)	103/1936 (5)	290/2481 (12)	128/1680 (8)	614/2441 (25)	53/1936 (3)	528/2441 (22)	58/2095 (3)	5/1672 (0)	77/1026 (8)	583/2209 (26)

aIL6: interleukin 6 inhibitors; aTNF: tumor necrosis factor alpha inhibitor; AZA: azathioprine; HCQ: hydroxychloroquine; MMF: mycophenolate; MTX: methotrexate;OCZ: ocrelizumab; PDN: prednisone (or equivalents); RTX: rituximab.

Supplementary Table 2: selected adverse reactions after the first dose of adenoviral-vectored and mRNA vaccines in patients with autoimmune diseases

	Boekel et al.		Cherian et al.	Allen-Philbey et al.	Studies with mRNA vaccines (pooled data)^
	ChAdOx1-nCoV-19	mRNA vaccines			
**Patients: N**	231	274	513	33	1788
**Patients vaccinated with adenoviral vector vaccines: n (%)**	231 (100)	274 (100)	447 (87)	29 (88)	0 (0)
**Allergic AEs**	4 (2)	0 (0)	ND	ND	0/1023 (0)
**Disease flares**	14 (6)	12 (4)	4 (1)	ND	36/1397 (3)
**Local pain**	89 (39)	107 (39)	128 (25)	23 (70)	871/1485 (59)
**Fatigue**	83 (36)	56 (20)	92 (18)	ND	251/1401 (18)
**Arthralgia/Arthritis**	31 (13)	18 (7)	ND	ND	50/1076 (5)
**Myalgia**	11 (5)	9 (3)	49 (10)	ND	169/1401 (12)
**Headache**	81 (35)	43 (16)	71 (14)	ND	247/1401 (18)
**Fever**	39 (17)	17 (6)	94 (18)	7 (21)	64/1401 (5)
**Chilling**	52 (23)	20 (7)	14 (3)	ND	80/1401 (6)
**Other**	34 (15)	38 (14)	ND	ND	104/1401 (7)

^: Connolly CM et al., Geisen U et al., Ramirez GA et al., Callejas-Rubio JL et al, Furer V et al., Boekel L et al., Mahil SK et al., Simon D et al., Braun-Moscovici Y et al., Damiani G et al.. ND: no data.

Supplementary Table 3: vaccination efficacy in patients treated with B-cell depleting agents

Ref.	Vaccine(s)	N	Mean / median time from last RTX (months)	Clinical endpoint (COVID-19)	Serological endpoint	Cellular response endpoint	Responders: N (%)		
							Clinical	Humoral	T-cellular
Achiron A et al	BNT162b2	44	4.9	No	Yes	No	ND	10/44 (23)	ND
Buttari F et al.	BNT162b2 and ChAdOx1 nCoV-19	2	2.5	No	Yes	No	ND	1/2 (50)	ND
Bonelli MM et al.	BNT162b2	5	5.7	No	Yes	Yes	ND	2/5 (40)	5/5 (100)
Ramirez GA et al.	BNT162b2	6	ND	Yes	No	No	6/6 (100)	ND	ND
Ruddy JA et al.	BNT162b2 and m-1372	19	ND	Yes	Yes	No	19/19 (100)	6/19 (32)	ND
Salviani C et al.	BNT162b2	2	6	No	Yes	No	ND	0/2 (0)	ND
Furer V et al.	BNT162b2	87	12	Yes	Yes	No	87/87 (100)	36/87 (41)	ND
Braun-Moscovici Y et al.	BNT162b2	48	7.6	No	Yes	No	ND	24/48 (50)	ND
Guerrieri S et al.	BNT162b2 and m-1372	16	5	No	Yes	No	ND	6/16 (38)	ND
Simon D et al. (2)	BNT162b2	8	3.1	No	Yes	Yes	ND	0/8 (0)	6/8 (75)
Total	NA	237	NA	3/10 (30)	9/10 (90)	2/10(20)	112/112 (100)	85/231 (37)	11/13 (85)

Supplementary Table 4: incidence of positive skin tests to vaccine excipient in patients with allergy history and incident allergy to anti-SARS-CoV-2 vaccines

Ref.	Vaccine(s)	Skin tests		General vaccinee population	Self-reported history of allergy	Confirmed history of allergy	Deemed at risk and tested	Additional tests	Additional patients with possible allergy to the first dose
Rojas-Pérez-Ezquerra P et al.	BNT162b2 and m-1372			ND	ND	131	131	0	0
		N of positive skin tests to the vaccine	0	NA	NA	0,0%	0,0%	NA	NA
		N of positive skin tests to PEG	2	NA	NA	1,5%	1,5%	NA	NA
		N of positive skin tests to PS80	0	NA	NA	0,0%	0,0%	NA	NA
		N of positive skin tests to trometamol	0	NA	NA	0,0%	0,0%	NA	NA
Paoletti G et al.	BNT162b2			4042	414	76	18	7	7
		N of positive skin tests to the vaccine	ND	NA	NA	NA	NA	ND	NA
		N of positive skin tests to PEG	1	0,02%	0,24%	1,32%	5,56%	1	14,29%
		N of positive skin tests to PS80	1	0,02%	0,24%	1,32%	5,56%	1	14,29%
		N of positive skin tests to trometamol	ND	NA	NA	NA	NA	ND	NA
Banerji A et al.	m1372, Ad26.COV2.S and BNT162b2			ND	ND	472	16	0	0
		N of positive skin tests to the vaccine	ND	NA	NA	NA	NA	NA	NA
		N of positive skin tests to PEG	1	NA	NA	0,21%	6,25%	NA	NA
		N of positive skin tests to PS80	ND	NA	NA	NA	NA	NA	NA
		N of positive skin tests to trometamol	ND	NA	NA	NA	NA	NA	NA
